# Spotlights on education and training in psychiatry: an international perspective

**DOI:** 10.1192/bji.2022.4

**Published:** 2022-05

**Authors:** Hussien Elkholy

**Affiliations:** Neurology and Psychiatry Department, Faculty of Medicine, Ain Shams University, Cairo, Egypt. Email: h.elkholy@med.asu.edu.eg

**Keywords:** Education and training, low- and middle-income countries, psychiatry, international medical graduates, research and publishing

## Abstract

Training and education must be frequently reviewed and developed to match the pace of rapid advances in psychiatry, preparing current and future psychiatrists to cope with the increasing amount of knowledge and skill required. In this, there is a need to ensure that training delivered internationally meets a common standard. This is essential not only to provide equivalent quality of care worldwide, but also to eliminate one of the obstacles faced by international medical graduates working abroad. Having the basic skills to publish work and viewpoints is essential in this era of rapid development and technological advancement to ensure sharing of knowledge and expertise.

Psychiatric education and training have progressed a great deal in recent decades. With the continuous development of investigations, discoveries and management plans, psychiatrists are being challenged by the increasing amount of knowledge they are expected to have. Besides up-to-date knowledge, psychiatrists of the future need to be prepared to adapt to the rapid advances in the field of mental health.^1^ Proper undergraduate psychiatric education is essential to allow future doctors to shape a healthy, non-stigmatising view of mental health issues, while also being able to incorporate knowledge from different medical specialty training. Additionally, knowing how to reach publication is essential to the sharing of knowledge and experience. This editorial gives an overview of three articles covering the special theme of education in psychiatry in this issue of *BJPsych International*.

## Undergraduate psychiatric education

The shortage of graduates choosing psychiatry as a career has been frequently raised. Medical students’ attitude towards psychiatry may improve if they have positive experiences of teaching, elective placements and exposure to psychiatric patients. Improving the quality of undergraduate education can support the process of destigmatisation of psychiatry as students can develop unbiased opinions regarding the specialty. Moreover, given the high prevalence of mental health problems in the general population, all doctors should have some knowledge and the basic skills to identify and address mental health problems.

Undergraduate psychiatric training varies across countries and, on occasion, even within the same country. Several unmet needs have been identified in the literature, including but not limited to restricted availability of educators, resources and appropriate methods. ‘Undergraduate psychiatric education: current situation and way forward’,^2^ the first article on our theme, discusses these points and offers examples for some possible improvements.

## International medical graduates and the challenges they face

Another challenging aspect is the lack of consensus on how undergraduate and postgraduate psychiatric education and training should be delivered (even within the same country), resulting in international medical graduates (IMGs) working abroad having to negotiate long tiring pathways to prove they are qualified. Despite the valuable service that IMGs provide to the healthcare of the countries they move to, this can be a significant barrier to career progression. It can be very frustrating as evidence suggests that where IMGs are given the opportunity, they are able to make significant contributions. Governments and institutions, alongside mentoring and peer support, may play an important role in overcoming the difficulties IMGs face. A comprehensive view of this situation can be obtained from the second article on our theme, titled ‘International medical graduates: challenges and solutions in psychiatry’.^3^

## The art of getting published

Research and publication are essential to the development of any science. The field of mental health is especially thirsty for more. However, many doctors find it very difficult to get their work published and do not have the proper mentorship and support to negotiate research avenues.

The concept of ‘publish or perish’ has been passed down the generations in academic and research fields. The pressure on researchers to have their work published in order to be promoted, or to even secure their job in the first place, makes the process repellent to some. This is unfortunate as, besides those who have dedicated their careers to research or academia, whether fully or partially, clinicians do have valuable experiences that need to be shared. Sadly, the skills required to achieve publication are not universally included in training programmes.

However, there is some simple advice that can help those interested in sharing their work or findings to get their publications accepted. In ‘Education in psychiatry: the art of getting published’,^4^ David Skuse, Editor of *BJPsych International*, sheds light on some techniques to help and common pitfalls to avoid. Hopefully, this article will help current and future researchers understand the process, and allow more clinicians to share their knowledge and findings and to have their voices heard.

## Data availability

Data availability is not applicable to this article as no new data were created or analysed in this study.

## References

[1] Bhugra D, Tasman A, Pathare S, Priebe S, Smith S, Torous J, The WPA–Lancet Psychiatry commission on the future of psychiatry. Lancet Psychiatry 2017; 4: 775–818. 28946952 10.1016/S2215-0366(17)30333-4

[2] Sampogna G, Elkholy H, Baessler F, Coskun B, Pinto da Costa M, Ramalho R, Undergraduate psychiatric education: current situation and way forward. BJPsych Int 2022: this issue. 10.1192/bji.2021.48 PMC9046837 35532467

[3] Pemberton M, Gnanapragasam S, Bhugra D. International medical graduates: challenges and solutions in psychiatry. BJPsych Int 2022: this issue. 10.1192/bji.2021.15 PMC9046820 35532419

[4] Skuse DH. Education in psychiatry: the art of getting published. BJPsych Int 2022: this issue. 10.1192/bji.2021.27 PMC9046821 35532466

